# Usefulness of an artificial neural network for a beginner to achieve similar interpretations to an expert when examining myocardial perfusion images

**DOI:** 10.1007/s10554-021-02209-z

**Published:** 2021-03-11

**Authors:** A. Chiba, T. Kudo, R. Ideguchi, M. Altay, S. Koga, T. Yonekura, A. Tsuneto, M. Morikawa, S. Ikeda, H. Kawano, Y. Koide, M. Uetani, K. Maemura

**Affiliations:** 1 Department of Cardiovascular Medicine, Nagasaki University Graduate School of Biomedical Sciences, Nagasaki, Japan; 2 Department of Radioisotope Medicine, Atomic Bomb Disease Institute, Nagasaki University, 1-7-1 Sakamoto, Nagasaki, 〒8528102 Japan; 3 Department of Radiology, Nagasaki University Hospital, Nagasaki, Japan; 4 Department of Cardiovascular Medicine, Nagasaki University Hospital, Nagasaki, Japan; 5 Department of Radiological Sciences, Nagasaki University Graduate School of Biomedical Sciences, Nagasaki, Japan; 6 Nagasaki Medical Education Center, Nagasaki University Hospital, Nagasaki, Japan; 7 Department of Cardiovascular Medicine, Nagasaki Memorial Hospital, Nagasaki, Japan

**Keywords:** Artificial neural network, Myocardial perfusion images, Artificial intelligence, Nuclear medicine, Cardiology imaging

## Abstract

This study examined whether using an artificial neural network (ANN) helps beginners in diagnostic cardiac imaging to achieve similar results to experts when interpreting stress myocardial perfusion imaging (MPI). One hundred and thirty-eight patients underwent stress MPI with Tc-labeled agents. An expert and a beginner interpreted stress/rest MPI with or without the ANN and the results were compared. The myocardium was divided into 5 regions (the apex; septum; anterior; lateral, and inferior regions), and the defect score of myocardial blood flow was evaluated from 0 to 4, and SSS, SRS, and SDS were calculated. The ANN effect, defined as the difference in each of these scores between with and without the ANN, was calculated to investigate the influence of ANN on the interpreters' performance. We classified 2 groups (insignificant perfusion group and significant perfusion group) and compared them. In the same way, classified 2 groups (insignificant ischemia group and significant ischemia group) and compared them. Besides, we classified 2 groups (normal vessels group and multi-vessels group) and compared them. The ANN effect was smaller for the expert than for the beginner. Besides, the ANN effect for insignificant perfusion group, insignificant ischemia group and multi-vessels group were smaller for the expert than for the beginner. On the other hand, the ANN effect for significant perfusion group, significant ischemia group and normal vessels group were no significant. When interpreting MPI, beginners may achieve similar results to experts by using an ANN. Thus, interpreting MPI with ANN may be useful for beginners. Furthermore, when beginners interpret insignificant perfusion group, insignificant ischemia group and multi-vessel group, beginners may achieve similar results to experts by using an ANN.

## Introduction

During the last 20 years, artificial intelligence (AI) and machine learning (ML), including artificial neural networks (ANN), have markedly developed. AI and ML may aid medical imaging-based diagnosis, not only in terms of the detection of disease, but also in management, reporting, and prognostication [1]. ANN is a computational model of ML based on the human brain. It has been found that ANN are powerful tools for pattern recognition, signal processing, image or speech data compression, and learning expert systems [2]. There are 6675 radiologists with specialty licenses in Japan, according to the Japan Radiological Society, but only 1317 radiologists have specialty licenses in nuclear medicine. In particular, the number of experts in cardiac nuclear medicine is very small. The interpretation of myocardial perfusion images (MPI) requires skilled expert reading, but there are not as many skilled experts in the interpretation of MPI as there are in the interpretation of computed tomography scans in Japan.

Recently, many studies have shown that AI and ML significantly improve the diagnostic accuracy not only in cardiology, but also in many other medical fields [3, 4]. However, there has not been sufficient research on the use of AI in cardiac nuclear medicine. Nuclear medicine imaging techniques, such as MPI, do not provide as much data as other imaging techniques; therefore, AI may be useful for inexperienced physicians working in radiology or cardiology when they interpret MPI. If the use of AI helps beginners to achieve similar interpretations to experts when examining MPI, it would help to compensate for a lack of human resources.

The purpose of this study was to examine whether a beginner in diagnostic cardiac imaging can achieve similar results to an expert when interpreting stress MPI by using an ANN.

## Methods

### Subjects

The subjects were 138 consecutive patients who underwent stress MPI (with Tc-labeled agents) at Nagasaki University Hospital between May 2014 and June 2015, including 52 patients (38%) with multivessel disease, 35 patients (25%) with a history of myocardial infarction, and 21 patients (15%) with both multivessel disease (MVD) and a history of myocardial infarction. MVD is defined as 2 and more coronary arteries with 75% and more stenosis evaluated by CT angiography (CTA) and/or coronary angiography (CAG). Of these 138 patients, 8 patients (6%) underwent only CTA, 61 patients (44%) underwent only CAG, and 26 patients (19%) underwent both CTA and CAG. Of 52 patients with MVD, 1 patient (2%) underwent CTA, 32 patients (62%) underwent CAG, and 19 patients (37%) underwent both CTA and CAG. An expert with over 20 years’ interpretation experience and a beginner with a few years’ interpretation experience interpreted stress MPI with/without software, which implemented ANN, and the results were compared. They interpreted randomly these MPI without any information except patients' age and sex. The patients’ characteristics are shown in Table 1.

**Table 1:** Demographics of patients that underwent stress myocardial perfusion imaging

n = 138	Mean ± SD (range), n (%)
Age (years)	70.6 ± 0.8 (36–87)
Sex (male)	94 (68.1%)
Height, weight (male)	164 ± 0.7 cm, 61.3 ± 1.2 kg
Body mass index (male)	22.7 ± 0.4 kg/cm^2^
Height, weight (female)	149.7 ± 1.0 cm, 48.8 ± 1.5 kg
Body mass index (female)	21.8 ± 0.6 kg/cm^2^
No. of vessels displaying	
≥ 75% stenosis (0, 1, 2, 3)	63:23:17:35 (MVD: 38%)
Hypertension	94 (68.1%)
Diabetes mellitus	53 (38.4%)
Dyslipidemia	93 (67.4%)
History of MI	35 (25.3%)
History of PCI/CABG	53 (38.4%), 18 (13%)
Only CTA	8 (6%)
Only CAG	61 (44%)
Both CTA and CAG	26 (19%)
LVEF (%) (QGS, stress)	67.4 ± 1.4
LVEF (%) (QGS, rest)	68.7 ± 1.0
LVEDV (ml) (QGS, stress)	31.8 ± 3.3
LVEDV (ml) (QGS, rest)	32.5 ± 2.2

*CABG* coronary artery bypass grafting, *MI* myocardial infarction,*MVD* multivessel disease, *PCI* percutaneous coronary intervention, *LVEF* left ventricle ejection fraction, *LVEDV* left ventricle end-diastolic volume, *QGS* quantitative gated SPECT, *CTA* CT angiography, *CAG* coronary angiography

### Imaging

The stress and rest MPI studies were performed using a 1-day stress-first protocol and about 1200 MBq of a ^99m^Tc-labeled myocardial perfusion agent (tetrofosmin or MIBI; divided into 300 MBq for the stress imaging and 900 MBq for the rest imaging). The indications and stress protocols followed the guidelines of the Japanese Circulation Society [5]. An adenosine stress test was performed with a standard continuous injection protocol, involving an injection rate of 0.12 mg·kg^−1^·min^−1^, in 136 patients (98.6%), and an exercise stress test, involving symptom-limited ergometer exercise, was conducted in 2 patients (1.4%). The end-points of the exercise stress test included significant symptoms (such as chest pain, dyspnea, or leg fatigue), the achievement of the target heart rate, electrocardiographic changes (ST depression, ST elevation, fatal arrhythmia, or blood pressure problems [very high pressure of > 250 mmHg or hypotension]). One hour after the injection of the tracer, the patients were imaged using a dual-headed single-photon emission computed tomography (SPECT) system, equipped with low-energy high-resolution collimators (e.cam Signature; Siemens Healthcare GmbH, Germany); a 180° arc; and a 16 frames/beat acquisition protocol. The acquisition energy level was set at 140 keV with a 20% window fitted for ^99m^Tc. All of the patients were instructed to refrain from eating food (breakfast) before the scans.

### Image interpretation and scoring

**Fig. 1:**
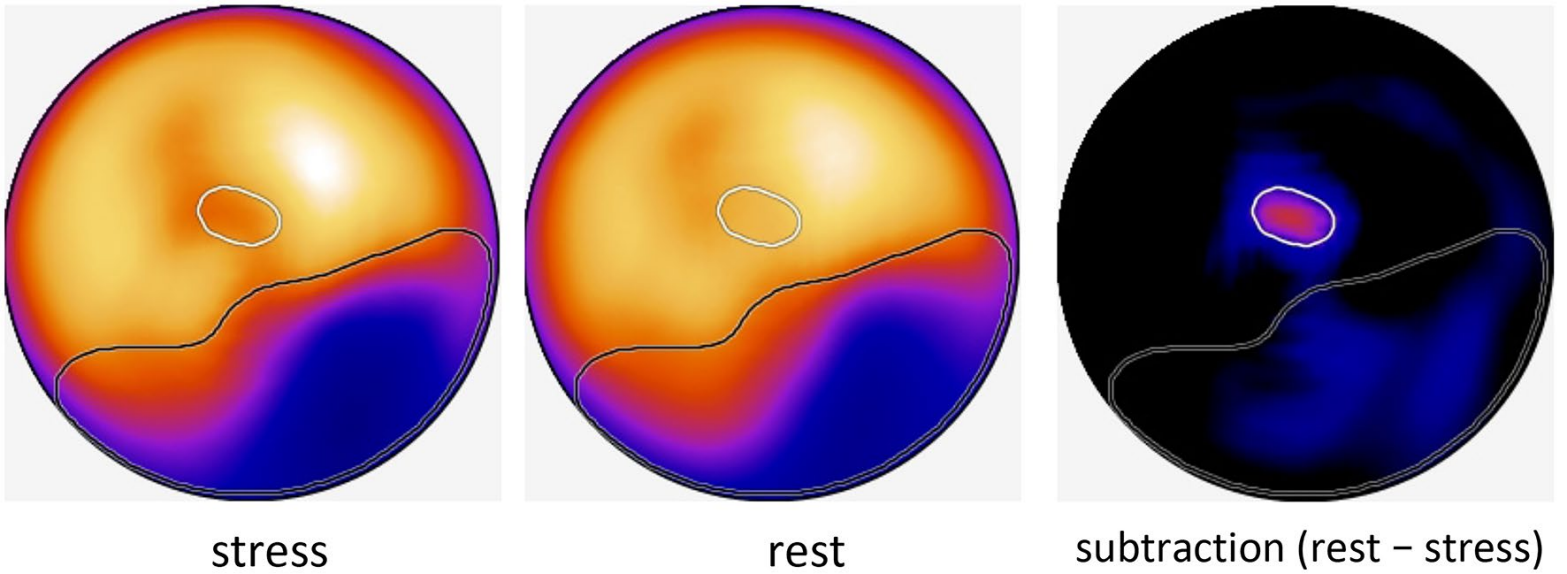
ANN analysis of stress MPI by cardioREPO. The region within the black line: the region exhibiting abnormal perfusion. The region within the white line: the ischemic region. The region within the black line, but not within the white line: a myocardial infarction [6]

The images were interpreted and scored in a medical image viewer, using the hospital’s Picture Archiving And Communication System (PACS) (Synapse; Fujifilm, Tokyo, Japan) with or without the diagnostic ANN software, (cardioREPO, version 1.1; Fujifilm Toyama Chemical, Tokyo, Japan). This software analyzes MPI via a ML system, which was trained using about 1000 patients’ images, and displays areas of abnormal stress perfusion and ischemic areas on a polarmap. The details of the method are described in Fig. 1 [6, 7]. Briefly, ANN software is trained with about 1000 patients’ stress polarmap and subtraction polarmap of stress and rest. When the users loaded stress and rest short axis images of each patient, the software automatically creates polarmaps of stress, rest and subtraction of stress and rest. Then calculate ANN value which is a probability of abnormal myocardium on stress polarmap which correspond to both infarction and ischemia. The program also draws white lines on the polarmap, indicating abnormal areas detected on subtraction polarmap which mainly correspond to ischemia. However, the interpretation of ischemia on this program is different from that with human interpretation, abnormal area indicated on black line and white line does note match with human interpretation always (for example, with human interpretation, ischemia is always included in the abnormal area on stress images because human interpret ischemia as abnormal on stress but normal on rest. However, ischemia on program is sometimes locates outside of stress abnormal area because ischemia is detected on the subtraction polarmap, not with the comparison of stress and rest polarmap).

Usually, a 17-segment model is used for myocardial perfusion scoring [8]. However, our study aimed to examine the effects of AI on the performance of beginners during image interpretation. To simplify the scoring for the beginner, the myocardium was divided into 5 regions; i.e., the apex; septum; and the anterior, lateral, and inferior regions [9]. The degree of abnormal perfusion distribution defined as defect score was classified from 0 to 4 (0; normal perfusion, 1; mild drop in perfusion, 2; moderate drop in perfusion, 3; severe drop in perfusion, 4: defect of perfusion). The sum total of these 5 regions measured on stress image was defined as summed stress score (SSS), that measured on rest image was defined as summed rest score (SRS), and the difference between SSS and SRS (SSS – SRS) was defined as summed difference score (SDS). Thus, high SSS and high SRS (= low SDS) corresponds to myocardial infarction, high SSS and low SRS (= high SDS) corresponds to myocardial ischemia and low SSS and low SRS (= low SDS) corresponds to normal myocardium. Max SSS in this study is 20 in our 5 regions model on the count. These parameters were obtained without help of the ANN once. Then after several weeks interval to ensure that previous interpretation would fade from the memory of interpreters, score parameters were again obtained with help of ANN display (Fig. 1) side by side to original image. Using this ANN polarmap display as additional information, both interpreters scored each image individually. Namely a beginner and an expert interpreted individually myocardial SPECT images and scored SSS, SRS, and SDS with only information of these patients’ sex and age without ANN. Polarmaps are like answers of these images, so they interpreted each segments model (horizontal long axis view, short axis view, vertical long axis view) without Polarmaps. To avoid influence on previous interpreting, after several day interval, they interpreted individually myocardial SPECT images with same manner but with help of ANN display. In order to investigate the influence of the ANN on the interpreters’ performance, the ANN effect was calculated as the difference in each score (SSS, SRS, or SDS) between with and without the ANN for both the beginner and expert. A larger ANN effect indicates that the findings were interpreted more accurately with than without the ANN.

We classified severity of abnormal perfusion into four categories (normal, mild, moderate and severe) with following manners. We classified SSS and SDS into four categories using classification used by Czaja et al. Czaia used 17 segment model and we used 5 segment model. Thus the number threshold used for the classification was converted according to this difference of segment number resulting SSS/SDS = 0 was classified as normal (or minimally abnormal), SSS/SDS = 1 was classified as minimally abnormal, SSS/SDS = 2 was classified as moderately abnormal and SSS/SDS = 3 or more was classified as significantly abnormal [10]. Patient with %ischemia exceeding 10% are believed to benefit from revascularization regardless of their left ventricular ejection fraction [11]. Thus in our analysis, SDS = 2 or more corresponds to %ischemia over 10%. We show Table 2 for help. When max SSS in our study is 20, it’s too small to classify 4 groups (normal, mild abnormal, moderate abnormal, severe abnormal). Therefore we classified 2 groups (insignificant perfusion group; SSS of 0 and 1, significant perfusion group; SSS of 2 and more) and compared them. In the same way, we classified 2 groups (insignificant ischemia group; SDS of 0 and 1, significant ischemia group; SDS of 2 and more) and compared them. Besides, we classified 2 groups (with normal vessels (no coronary artery with 75% and more stenosis) and with multi-vessels (2 and more coronary arteries with 75% and more stenosis) and compared them.

**Table 2:** Citation and alteration from [10] (when max SSS = 20)

SSS	SS%	SDS	Result
0	< 5	0	Normal or minimally abnormal
1	5–9	1	Mildly abnormal
2	10–14	2	Moderately abnormal
3-	> 14	3-	Significantly abnormal

Since ANN is not given in segmental values, how the ANN results were incorporated into final interpretation can be explained as following.

When we interpret the image with ANN, ANN analysis presented on the polarmap (Fig. 1) is displayed side by side on the display with slice images which we interpreted without ANN. Using this ANN polarmap display as additional information, we interpret the slice images.

### Statistics

All data are expressed as mean ± standard deviation (SD) values. The significance of differences was examined by one-way analysis of variance with the F test or paired t-test. P-values of < 0.05 were considered to be significant. All statistical analyses were performed using the JMP 10.0.2 software.

All procedures performed in studies involving human participants were in accordance with the ethical standards of the institutional research committee and with the 1964 Declaration of Helsinki and its later amendments. All clinical data were completely anonymized and processed at Nagasaki University Hospital. This study was approved by Nagasaki University Hospital Clinical Research ethics committee (Approval No. 15072762).

## Results

### Interpretation of images

The results are shown in Table 3. The ANN effect for all 138 patients was smaller for the expert than for the beginner (SSS: − 0.49 vs. − 1.23, p < 0.0001; SRS: − 0.34 vs. − 0.88, p = 0.0003; SDS: − 0.15 vs. − 0.36, p = 0.0128, respectively). The ANN effects for all 138 patients on SSS, SRS, and SDS were negative for both interpreters, which indicates that they had lower scores with than without the ANN; i.e. their diagnostic approach became more conservative when they were using the ANN. The absolute ANN effect value was lower in the expert than in the beginner, which means that the abovementioned effect was larger in the beginner. When the scores obtained by the expert with the ANN were defined as standard scores, the SSS and SRS scores of the beginner were closer to the standard scores with than without the ANN.

**Table 3:** Results

ANN effect	Expert	Beginner	p-value
SSS	− 0.49 ± 0.08	− 1.23 ± 0.15	< 0.0001
SRS	− 0.34 ± 0.07	− 0.88 ± 0.13	0.0003
SDS	− 0.15 ± 0.06	− 0.36 ± 0.08	0.0128

*ANN* artificial neural network, *SSS* summed stress score, *SRS* summed rest score, *SDS* summed difference score

The more detailed results are shown in Table 4. The ANN effect for 78 patients (insignificant perfusion group, SSS = 0 and 1) was smaller for the expert then for the beginner (SSS: − 0.27 vs. − 1.28, p < 0.0001; SRS: − 0.09 vs. − 0.88, p < 0.0001; SDS: − 0.18 vs. − 0.40, p = 0.0185, respectively). The ANN effect for 116 patients (insignificant ischemia group, SDS = 0 and 1) was smaller for the expert than for the beginner (SSS: − 0.30 vs. − 1.28, p < 0.0001; SRS: − 0.30 vs. − 0.97, p < 0.0001; SDS:0 vs. − 0.30, p = 0.0003, respectively).

**Table 4:** Precise results

ANN effect	Expert	Beginner	p-value
Insignificant perfusion group (SSS = 0 and 1)			
SSS	− 0.27 ± 0.55	− 1.28 ± 1.39	< 0.0001
SRS	− 0.09 ± 0.43	− 0.88 ± 1.28	< 0.0001
SDS	− 0.18 ± 0.50	− 0.40 ± 0.80	0.0185
Significant perfusion group (SSS = 2 and more)			
SSS	− 0.78 ± 1.19	− 1.17 ± 2.09	0.2060
SRS	− 0.67 ± 1.02	− 0.87 ± 1.93	0.4631
SDS	− 0.12 ± 0.98	− 0.30 ± 1.08	0.2067
Insignificant ischemia group (SDS = 0 and 1)			
SSS	− 0.30 ± 0.77	− 1.28 ± 1.66	< 0.0001
SRS	− 0.30 ± 0.78	− 0.97 ± 1.55	< 0.0001
SDS	0 ± 0.62	− 0.30 ± 0.87	0.0003
Significant ischemia group (SDS = 2 and more)			
SSS	− 1.50 ± 1.01	− 1.00 ± 2.05	0.3732
SRS	− 0.55 ± 0.86	− 0.36 ± 1.71	0.7218
SDS	− 0.95 ± 0.84	− 0.64 ± 1.18	0.2162
Normal vessels group (no coronary artery with 75% and more stenosis)			
SSS	− 0.49 ± 0.69	− 0.95 ± 1.75	0.0623
SRS	− 0.29 ± 0.68	− 0.73 ± 1.52	0.0390
SDS	− 0.21 ± 0.63	− 0.22 ± 0.83	0.8802
Multi- vessels group (2 and more coronary arteries with 75% and more stenosis)			
SSS	− 0.49 ± 1.08	− 1.47 ± 1.67	< 0.0001
SRS	− 0.39 ± 0.88	− 1.00 ± 1.64	0.0031
SDS	− 0.11 ± 0.83	− 0.47 ± 0.99	0.0028

*ANN* artificial neural network, *SSS* summed stress score, *SRS* summed rest score, *SDS* summed difference score, *MVD* multi-vessel disease

The ANN effect for 60 patients (significant perfusion group, SSS = 2 and more) was no significant between the expert and the beginner (SSS: − 0.78 vs. − 1.17, p = 0.2060; SRS: − 0.67 vs. − 0.87, p = 0.4631; SDS: − 0.12 vs. − 0.30, p = 0.2067, respectively).

The ANN effect for 22 patients (significant ischemia, SDS = 2 and more) was no significant between the expert and the beginner (SSS: − 1.50 vs. − 1.01, p = 0.3732; SRS: − 0.55 vs. − 0.36, p = 0.7218; SDS: − 0.95 vs. − 0.64, p = 0.2162, respectively).

## Discussion

Our results indicate that an ANN reduced the image interpretation gap between an expert and beginner. In a previous study, neural network software showed sensitivity of 90% and specificity of 85% for detecting myocardial ischemia, which were superior to the sensitivity and specificity of a computer-assisted diagnostic system (the Emory Cardiac Toolbox). In addition, a decision support system based on neural networks achieved interpretations that were more similar to those of experienced clinicians than those achieved by a conventional automated quantification software package [9]. Our study did not compare multiple methods, but rather evaluated the additional effect of using AI to aid image interpretation. However, both studies showed that AI improves the interpretation of MPI.

Our results regarding the skill effect showed that the SDS scores of the beginner were lower than those of the expert when the ANN was used. If the expert’s scores are taken as standard scores, this finding indicates that the beginner made false-negative interpretations of ischemia when using the ANN. In a study by Nakajima, when the ANN threshold was set at 0.5, the ANN interpreted regions without ischemia perfectly [12]. However, it missed some cases that were interpreted as ischemia by the consensus reading. This was especially common in cases of mild ischemia or mild infarctions. These findings indicate that compared with the expert consensus the ANN produced false-negatives for ischemia, which may explain our results. If a beginner interpreted images as suggested by such an ANN, but an expert was able to interpret them correctly, the beginner would produce false-negatives for ischemia when using the ANN. However, the interpretations of a single expert will not always be correct. Our results showed that even experts can change their interpretations of images when using ANN although the magnitude of the changes made by the expert was smaller than that of the changes made by the beginner. In a previous study, images were interpreted using an ANN and re-evaluated by 3 experts, and differences were found in 53 of 200 cases. This disagreement was related to small or mild perfusion defects, which indicates that the interpretations of the experts may have fluctuated or even been wrong [13]. An ANN may be useful for standardizing image interpretation, even for experts, which may explain why the scoring by the expert was still affected by the use of the ANN.

We did not compare the image interpretation using ANN with other method such as coronary stenosis etc. As described in previous study by Nakajima et al. the aim of this study is whether this software can help to be close to expert interpretation, not be close to detect true ischemia. As noted on the method, ANN was trained with expert reading and not including detailed clinical information such as FFR, coronary flow reserve, myocardial perfusion etc. Thus theoretically, the ANN can be close to but cannot exceed expert.

We used an ANN to aid image analysis/interpretation. Arsanjani et al. showed that the LogitBoost method, which is another type of ML, exhibited almost the same accuracy when interpreting MPI as expert readers [14]. Our findings are similar.

## Limitations

This study involved an expert and a beginner in cardiac nuclear medicine and was performed at Nagasaki University. Although we should have included a few experts and a few beginners to obtain sufficient data, there was only one expert in cardiac nuclear medicine in Nagasaki Prefecture.

In addition, we used a simplified 5-segment model instead of the conventional 17-segment model for scoring. This narrowed the range of scores (for 5 segments, the maximum value is 20, whereas for 17 segments the maximum value is 68), which may have reduced the statistical power of the study. However, a previous study, which analyzed a neural network support system, also used a 5-segment model. Thus, we consider that this 5-segment model did not markedly affect our results [9].

Strictly speaking, we should compare our study results with gold standard, but our study target is to achieve going beginners' interpretation with AI up to human experts' that. So we didin't do it in this study [7].

## Conclusions

When using an ANN, when interpreting MPI of insignificant perfusion group and insignificant ischemia group, beginners may achieve similar imaging interpretations to experts in cardiac nuclear medicine. Furthermore, ANN systems may be useful for obtaining a second opinion, particularly when physicians are inexperienced at interpreting nuclear cardiology imaging.

## Data Availability

The datasets generated during and/or analyzed during the current study are available from the corresponding author on reasonable request.

## Abbreviations

MPI: Myocardial perfusion imaging
AI: Artificial intelligence
ANN: Artificial neural network
SSS: Summed stress score
SRS: Summed rest score
SDS: Summed difference score
ML: Machine learning
